# PTEN Hopping on the Cell Membrane Is Regulated via a Positively-Charged C2 Domain

**DOI:** 10.1371/journal.pcbi.1003817

**Published:** 2014-09-11

**Authors:** Masato Yasui, Satomi Matsuoka, Masahiro Ueda

**Affiliations:** 1 Laboratories for Nanobiology, Graduate School of Frontier Biosciences, Osaka University, Suita, Osaka, Japan; 2 Laboratory for Cell Signaling Dynamics, QBiC (Quantitative Biology Center), RIKEN, Suita, Osaka, Japan; 3 Laboratory of Single Molecule Biology, Department of Biological Sciences, Graduate School of Science, Osaka University, Toyonaka, Osaka, Japan; University of Colorado, United States of America

## Abstract

PTEN, a tumor suppressor that is frequently mutated in a wide spectrum of cancers, exerts PI(3,4,5)P_3_ phosphatase activities that are regulated by its dynamic shuttling between the membrane and cytoplasm. Direct observation of PTEN in the interfacial environment can offer quantitative information about the shuttling dynamics, but remains elusive. Here we show that positively charged residues located in the cα2 helix of the C2 domain are necessary for the membrane localization of PTEN via stable electrostatic interactions in *Dictyostelium discoideum*. Single-molecule imaging analyses revealed that PTEN molecules moved distances much larger than expected had they been caused by lateral diffusion, a phenomenon we call “hopping.” Our novel single-particle tracking analysis method found that the cα2 helix aids in regulating the hopping and stable-binding states. The dynamically established membrane localization of PTEN was revealed to be essential for developmental processes and clarified a fundamental regulation mechanism of the protein quantity and activity on the plasma membrane.

## Author Summary

The plasma membrane is a major chemical reaction field in living cells, and the molecular mechanisms of protein-membrane interactions are important for many cellular functions. In this report, we have discovered that the PTEN protein, which transits between the cytoplasm and membrane, hops along the plasma membrane of living cells. We tracked individual PTEN molecules on the membrane by single molecule imaging and analyzed the hopping behavior by developing a novel analysis method, which measures the rebinding probability of membrane-bound proteins after detaching from the membrane. We found that positively charged amino acids in the C2 domain of PTEN, which were reported to be important for its phosphatase activity on the membrane, are required to suppress excessive hopping and stabilize PTEN membrane binding. The stable electrostatic interactions localize PTEN to the plasma membrane and play an indispensable role in regulating the size of the multicellular structures formed under a starving environment. Our results suggest electrostatic interactions between the protein and membrane regulate protein quantity and activity.

## Introduction

Molecular reactions on the plasma membrane are responsible for various cellular functions. The inner surface of the cell membrane is covered with a negatively charged layer up to 20 nm thick that arises from the polar head groups of anionic phospholipids [1]. Cytoplasmic proteins that possess positively charged patches on their surface due to cationic amino acid residues are thus electrostatically attracted to the membrane. Such electrostatic interactions provide an essential mechanistic basis for the translocation of cytoplasmic proteins to the membrane, which is usually mediated via domains or motifs of the proteins including the C2 and pleckstrin homology (PH) domains [2], [3]. Based on the specificity of the interaction, these domains and motifs can serve as tags that direct the proteins toward the location of their functions.

**Figure 1:**
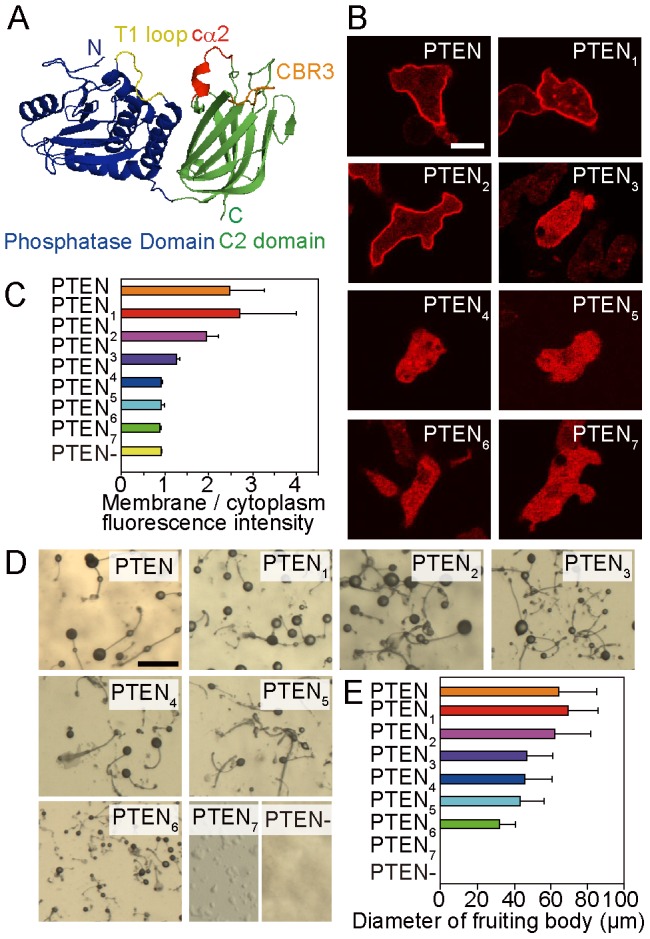
Phenotype of wild-type PTEN and PTEN*_i_* mutants (*i* = 1,2,…,7). (A) Human PTEN crystal structure (residues 14–351) [8]. PTEN*_i_* mutants had different positive charges in the cα2 helix. Regions colored in green and blue show the C2 domain and phosphatase domain, respectively. The red, yellow, and orange regions show the cα2 helix, T1 loop, and CBR3 loop, respectively. The PBM at the N-terminus (residues 1–13) and 24 residues in the C2 domain (residues 282–312) are not shown. The upper side of the structure faces the membrane. (B) Fluorescence images of *Dictyostelium discoideum* cells expressing wild-type PTEN or PTEN mutants. PTEN was labeled with TMR via HaloTag (PTEN-Halo-TMR). Images were captured by confocal microscopy. Scale bar, 5 µm. (C) The ratio of the plasma membrane and cytoplasm fluorescence intensities. (D) Images of fruiting bodies formed by wild-type cells or *pten*-null cells expressing wild-type PTEN or PTEN mutants. Scale bar, 500 µm (E) The diameter of the sorus in the fruiting bodies. Data are mean +/− SD.

Phosphoinositides and their surrounding electrostatic environments serve as essential mediators for a large variety of fundamental cellular functions. One example is phosphatidylinositol-3,4,5-trisphosphate (PI(3,4,5)P_3_), which activates downstream signaling pathways for cell growth and survival and whose unconditional activation causes cancer [4]. PI(3,4,5)P_3_ signaling can be antagonized by the tumor suppressor PTEN (phosphatase and tensin homologue deleted from chromosome 10), which dephosphorylates 3-phosphoinositides by dynamic shuttling between the membrane and cytoplasm [5]–[7]. Several regions of PTEN have been identified as necessary for the membrane interaction: the phosphatidylinositol-4,5-bisphosphate (PI(4,5)P_2_)-binding motif (PBM) at the N-terminus, the catalytic site and T1 loop in the middle phosphatase domain and cationic patches including the cα2 helix and CBR3 loop in the C-terminal C2 domain (Fig. 1A) [8]. While a point mutation can compromise substrate binding ability without significantly affecting membrane localization, substitutions of basic amino acids in the PBM, T1 loop, CBR3 loop, or cα2 helix decrease the affinity for vesicles containing anionic phospholipids such as phosphatidylserine (PS) and PI(4,5)P_2_ [8]–[11]. Therefore, the localization of PTEN to the membrane is likely regulated via electrostatic interactions with these anionic phospholipids.

Except for the cα2 helix, the above regions have already been shown indispensable for membrane localization in living cells. For example, the CBR3 loop seems to be masked in cultured mammalian cells by a negatively charged C-terminal tail due to multiple phosphorylations, which prevent PTEN from being recruited to the membrane [7], [12]–[14]. A mutation discarding positive charges on the T1 loop also alters the intracellular localization of PTEN from the membrane to the cytoplasm [10]. PBM significantly affects the intracellular localization of PTEN, causing *Dictyostelium* PTEN to be constitutively localized at the membrane due to the lack of a region that corresponds to the C-terminal tail of human PTEN [7], [9], [15]. Comparatively little is known about how the cα2 helix contributes to membrane localization in living cells. PTEN with the M-cα2 mutation (327-A-A-G-A-D-A-A-N-A-335) shows reduced affinity for phospholipid vesicles [8]. Owing to this property, although it retained phosphatase activity towards water-soluble PI(3,4,5)P_3_, the mutant failed to suppress the growth of glioblastoma cells. Since tumor derived mutations are found in the cα2 helix, novel insights into the membrane localization mechanism of PTEN might be obtained by studying the role of the cα2 helix in living cells [8].

Direct observation by single-molecule imaging has become a standard technique for analyzing the diffusive movements of molecules on the membrane and their shuttling between the membrane and cytoplasm. By observing fluorescently labeled molecules under total internal reflection fluorescence microscopy (TIRFM), the behaviors of molecules can be visualized on the basal cell membrane [16]. From the trajectories of the molecular movement on the membrane, spatiotemporal properties of the membrane interaction such as diffusion mobility and lifetime can be analyzed [17], [18]. These studies have stimulated the development of novel statistical analysis methods that can derive the mechanistic underpinnings of the molecular behaviors, leading to, for example, the finding of anomalous diffusion of biological molecules on the membrane [19], [20]. Recently, more complicated molecular behaviors at the membrane surface due to the electrostatic characteristics of the surface layer have been revealed. Falke and coworkers, based on studies of the PH domain of General Receptor for Phosphoinositides 1 (GRP1) binding to lipid vesicles in vitro, proposed an electrostatic search mechanism in which nonspecific adsorption to anionic phospholipids enhances the bulk on-rate of the specific binding to PI(3,4,5)P_3_ [21]. Their subsequent single molecule studies of the PH domain bound to supported lipid bilayers in vitro revealed that the protein often exhibits rebinding to the membrane, yielding long-range electrostatic “jumps”, instead of diffusing away into the solution [22]. They further proposed this same electrostatic search mechanism would be important in cells [21], [22], although this prediction has not yet been tested.

In the present study, the role of the cα2 helix in localizing PTEN to the plasma membrane was investigated by single molecule imaging, revealing that interactions with the membrane appear to be stabilized by positively charged residues on the cα2 helix. Moreover, we discovered PTEN molecules rebinding to the plasma membrane without diffusing away to the cytoplasm, as if they hopped along the membrane. A novel analysis method was developed to calculate the probability of hopping. The analysis results suggest that PTEN may adopt a specific state in which hopping is enhanced, and that the cα2 helix seems to be responsible for suppressing excessive hopping. Our analysis suggests the possibility that electrostatic interactions via the cα2 helix are utilized to not only stabilize membrane interactions but to also search the substrate on the membrane.

## Results

### Membrane localization of PTEN depends on the cα2 helix in the C2 domain

To examine the role electrostatic interactions in the cα2 helix have in regulating the amount of PTEN on the membrane, we constructed seven PTEN mutants with different numbers of positively charged amino acids. The mutants, which we named PTEN*_i_* (*i* = 1,2,…,7), had basic amino acids substituted into neutral ones so that the positive charge gradually decreased from PTEN_1_ to PTEN_7_ (Table 1). PTEN_7_ shares the same amino acid sequence as M-cα2 [8]. The mutant PTENs were tagged with HaloTag protein, and the intracellular localization was visualized in living *Dictyostelium discoideum* cells by labeling with tetramethylrhodamine. Wild-type PTEN localized on the plasma membrane in *Dictyostelium* wild-type cells (Fig. 1B), as reported previously [15]. However, the intracellular localization of the mutant was dependent on the number of amino acid substitutions, showing a gradual shift from the plasma membrane to the cytoplasm as the number increased. Fig. 1C shows the plasma membrane-to-cytoplasm ratio of averaged fluorescence intensity, indicating that the membrane localization critically dropped from PTEN_3_ and PTEN_4_. Therefore, a positively charged cα2 helix in the C2 domain, which most likely interacts with negatively charged membrane phospholipids, seems to be an essential factor regulating the amount of PTEN on the plasma membrane.

**Table 1:** Amino acids and primer sequences of PTEN mutants.

Name	Amino acid	Primer
PTEN(Human)	KANKDKANR	-
PTEN	KAHKDKNHK	gagtggtctcgataaagcacacaaagataaaaaccataaagctttcccagagg
PTEN_1_	KAHKDKNNK	gagtggtctcgataaagcacacaaagataaaaacaataaagctttcccagagg
PTEN_2_	KAHKDKANK	gagtggtctcgataaagcacacaaagataaagcaaataaagctttcccagagg
PTEN_3_	KAGKDKANK	gagtggtctcgataaagcaggtaaagataaagcaaataaagctttcccagagg
PTEN_4_	KAGKDKANA	gagtggtctcgataaagcaggtaaagataaagcaaatgcagctttcccagagg
PTEN_5_	KAGKDAANA	gagtggtctcgataaagcaggtaaagatgcagcaaatgcagctttcccagagg
PTEN_6_	KAGADAANA	gagtggtctcgataaagcaggtgcagatgcagcaaatgcagctttcccagagg
PTEN_7_ (M-cα2)	AAGADAANA	gagtggtctcgatgcagcaggtgcagatgcagcaaatgcagctttcccagagg

Underscores mark amino acids with positive charges.

The mutations critically affected the size of the multicellular fruiting bodies that formed after the deprivation of a food source. PI(3,4,5)P_3_ phosphatase activity of PTEN is indispensable for the intracellular communication mediated by cAMP, which is utilized to aggregate up to a hundred thousand cells before entering multicellular developmental stages. Consistently, *pten*-null cells did not aggregate or form fruiting bodies. Fig. 1D shows the fruiting body of *pten*-null cells expressing PTEN*_i_*. PTEN_7_ could not complement the aggregation-minus phenotype of the null cells. The diameter of the sorus of the fruiting body was correlated with the degree of membrane localization of PTEN*_i_* (Fig. 1E). Therefore, PTEN membrane localization mediated via the cα2 helix is critical for the size regulation and progression of multicellular development.

### Membrane interactions are stabilized due to positively charged residues

**Figure 2:**
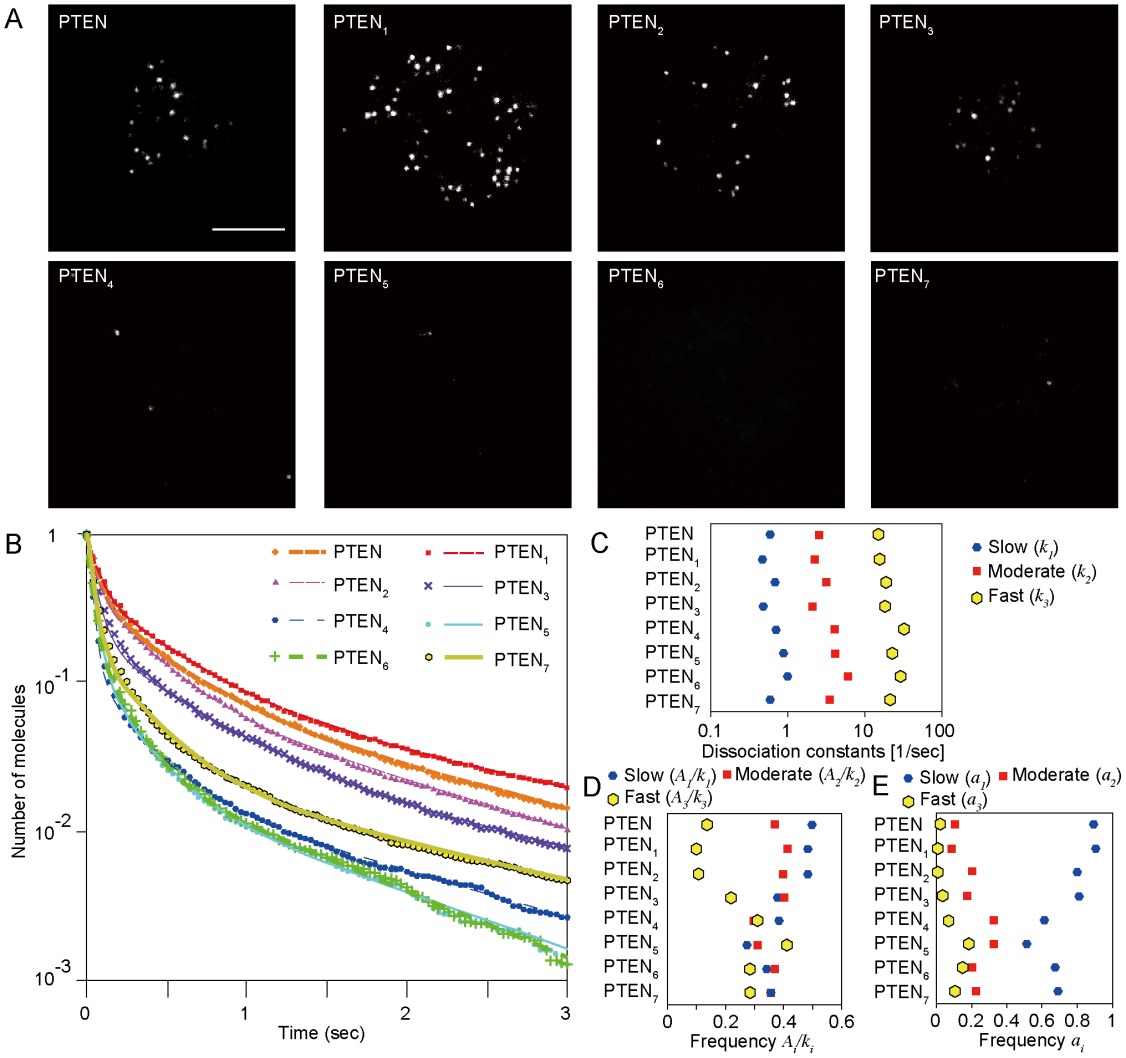
Single-molecule imaging of wild-type PTEN and PTEN*_i_* mutants. (A) Images of cells expressing wild-type PTEN or PTEN*_i_* mutants labeled with TMR captured under TIRFM. Scale bar, 5 µm. (B) The numbers of PTEN-Halo-TMR and PTEN*_i_*-Halo-TMR molecules that remained bound to the membrane are plotted against time after membrane association. Lines are three-component exponential fits (Eq. 1). The cumulative plots were obtained from 16,088 molecules in 8 cells (wild-type PTEN), 12,164 molecules in 8 cells (PTEN_1_), 9,022 molecules in 7 cells (PTEN_2_), 11,386 molecules in 8 cells (PTEN_3_), 12,406 molecules in 8 cells (PTEN_4_), 11,079 molecules in 8 cells (PTEN_5_), 10,822 molecules in 7 cells (PTEN_6_) and 20,683 molecules in 8 cells (PTEN_7_). (C, D) Dissociation constants *k*_1–3_ (C) and frequencies *A*_1–3_/*k*_1–3_ (D) of PTEN*_i_* mutants obtained from the fitting in (B). Estimated parameters are shown in Table 2. (E) Frequency of slow, moderate and fast diffusion mobility states, *a_i_*, from the displacement distribution analysis in Fig. S3.

In order to examine how these residues are involved in the membrane interaction at the molecular level, we observed the behavior of single PTEN molecules on the basal membrane under objective-type TIRFM. Single molecules were visualized as individual fluorescent spots when they were bound to the membrane, as shown in typical snapshots for each PTEN mutant (Fig. 2A and Fig. S1). The number of membrane-bound molecules of PTEN_4–7_ was lower on average than that of wild-type PTEN and PTEN_1–3_ (Movie S1), which argues the cα2 helix regulates the membrane localization of PTEN. As seen in the movies, fluorescent spots from PTEN_4–7_ molecules seem short-lived relative to those of the other PTEN molecules. The time duration of the membrane interaction was measured for all single molecules observed in the movies. In Fig. 2B, the number of molecules that remained bound to the membrane was counted every 33 msec beginning from the onset of membrane association, normalized by the total number of molecules and plotted against time [23], [24]. The decay rate of the number of molecules on the membrane, which corresponds to the membrane dissociation rate, varied depending on the number of mutations. Wild-type PTEN and PTEN_1_ displayed the slowest membrane dissociation, and PTEN_5_ and PTEN_6_ displayed the fastest. Thus, the kinetics of membrane dissociation was dependent on the number of positively charged residues in the cα2 helix. The curves were fitted by an exponential function with *n* components,
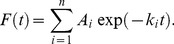
(1)


The fitting was better when assuming three components (Fig. 2B, Table 2) rather than two (Fig. S2, Table S1). Therefore, we concluded that PTEN adopts at least three states with different dissociation kinetics on the membrane at a frequency proportional to *A_i_*/*k_i_* (Table 2). The dissociation state with the slowest rate constant, *S*_1_, tended to show a decrease in frequency with increasing neutral charge, but constant *k*_1_ (Fig. 2C, D). On the other hand, the state with a moderate rate constant, *S*_2_, had a relative constant frequency but a slightly increasing *k*_2_ with increasing neutral charge. The state with the fastest rate constant, *S*_3_, showed an increase in both frequency and *k*_3_ with increasing neutral charge. Similarly, lateral diffusion during membrane interactions most likely consisted of three different diffusion coefficients based on fitting the displacement distribution to a probability density function (Table 3, Table S2, Fig. S3),
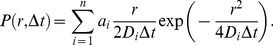
(2)Here, Δ*t* = 33 msec. Thus, there exist three mobility states. The frequency of each mobility state, *S′_i_*, decreased with increasing neutral charge for the lowest mobility state and increased for the moderate and fastest mobility states, indicating that the mobility and dissociation states, *S′_i_* and *S_i_*, were correlated (Fig. 2D, E). Therefore, the positive charge in the cα2 helix most likely stabilizes the membrane interaction of PTEN by suppressing dissociation. These results suggest that the kinetics of membrane dissociation is regulated by electrostatic interactions and that differences in the interaction strength affects the amount of PTEN on the membrane. Additionally, the charged state of the cα2 helix seems to correlate with dynamic transitions between states.

**Table 2:** Dissociation rate constants of wild-type PTEN and PTEN mutants in the three-component model.

Name	*A*_1_	*A*_2_	*A*_3_	*k*_1_ [1/sec]	*k*_2_ [1/sec]	*k*_3_ [1/sec]	*A*_1_ */k*_1_ [%]	*A*_2_ */k*_2_ [%]	*A*_3_ */k*_3_ [%]
PTEN	0.090	0.294	0.616	0.6	2.6	15.6	50	37	13
PTEN_1_	0.088	0.342	0.570	0.5	2.3	15.8	49	41	10
PTEN_2_	0.092	0.348	0.560	0.7	3.2	19.5	49	40	21
PTEN_3_	0.039	0.175	0.786	0.5	2.1	17.6	38	40	32
PTEN_4_	0.023	0.101	0.876	0.7	4.0	32.8	39	30	31
PTEN_5_	0.022	0.118	0.860	0.9	4.3	23.5	28	31	41
PTEN_6_	0.031	0.197	0.773	1.0	5.9	29.6	34	37	29
PTEN_7_	0.027	0.158	0.816	0.6	3.5	22.5	36	36	29

Parameters obtained from fitting the data in Fig. 2B with Eq. 1 (*n* = 3). The relative frequency of PTEN adopting each state was calculated from *A_i_*/*k_i_*.

**Table 3:** Diffusion constants of wild-type PTEN and PTEN mutants in the three-component model.

Name	*a*_1_	*a*_2_	*a*_3_	*D*_1_ [µm^2^/s]	*D*_2_ [µm^2^/s]	*D*_3_ [µm^2^/s]	*ε* [nm]
PTEN	0.889	0.102	0.020	0.017	0.084	0.705	21
PTEN_1_	0.907	0.092	0.010	0.022	0.100	1.338	18
PTEN_2_	0.798	0.206	0.011	0.025	0.081	1.019	16
PTEN_3_	0.810	0.176	0.032	0.021	0.081	0.920	25
PTEN_4_	0.617	0.328	0.067	0.005	0.036	0.728	32
PTEN_5_	0.515	0.326	0.187	0.012	0.071	0.655	37
PTEN_6_	0.679	0.198	0.145	0.013	0.067	0.658	36
PTEN_7_	0.690	0.226	0.105	0.013	0.086	0.666	34
PTEN_4_+Latrunculin A	0.521	0.268	0.262	0.006	0.056	0.776	45

Parameters obtained from fitting the data in Fig. S3 with Eq. 2 (*n* = 3). The observable lowest *D* was *ε* ^2^/Δ*t*, where *ε* is the standard deviation of the measurement error calculated from the mean square displacement of the trajectories [20].

### PTEN molecules “hop” on the cell membrane

**Figure 3:**
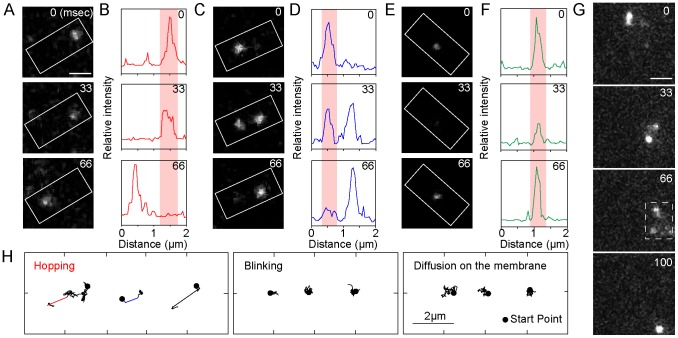
PTEN molecules hopping on the cell membrane. (A, B) Hopping occurred between the second and third frames. (C, D) Hopping occurred within the second frame. (E, F) Blinking. (A, C, E) Images of single molecules. (B, D, F) Fluorescence intensity profiles along the long axis of the rectangles shown in A, C and E. (G) Cloud-like fluorescence (dotted rectangle at *t* = 66 msec) of hopping molecules. Numbers in the upper right of each panel are time in milliseconds. Scale bar, 1 µm. (H) Trajectories of molecules showing hopping, blinking and lateral diffusion. Thick lines show hopping displacements. Colors in the trajectories indicate the moments of hopping shown in (B) and (D).

When we observed single molecules of PTEN_4_, we noticed that they often displayed an extraordinary large instantaneous displacement (Fig. 3). Fig. 3A shows a fluorescence spot in the movie that vanished at 33 msec, but had another spot emerge in the next movie frame at 66 msec. The distance between the two spots was longer than what is typically expected for a molecule undergoing lateral diffusion in that time. This large distance can be explained either by the two spots being one common fluorescent molecule or two distinct fluorescent molecules. That the vanishing and emerging fluorescent spots occurred anti-correlatively further argues the two are from the same molecule. There were also moments when two fluorescent spots that appeared had dimmer intensities than the single spots either before or after that moment (Fig. 3C). We sometimes also observed cloud-like fluorescent spots that had larger diameters and fainter intensities than average, which indicates that PTEN molecules can adopt an unusually fast diffusion mode (Fig. 3G). All these phenomena are clearly distinguished from fluorophores that blink and exhibit a much smaller displacement (Fig. 3E, H). We define these unexpectedly long displacements as hops by the molecule.

### Novel analysis method for hopping molecules in living cells

To validate that PTEN molecules hop on the membranes, we examined the probability that the fluorescent spots before and after hopping came from the same fluorescent molecule. For this purpose, the statistical analysis method proposed by Knight *et al*. was modified so that it could be applied to single molecules observed in living cells [22]. The analysis is based on a comparison between the frequency of spot emergence in a cell and the frequency of cytoplasmic molecule recruitment estimated from the data. When the former exceeds the latter, the emerged spots were deduced to most likely include molecules that hopped. When statistically estimating the recruitment frequency by *in vivo* single-molecule imaging, we incorporated two amendments to improve the accuracy: the effects of photo-bleaching and limitations in the observation area.

**Figure 4:**
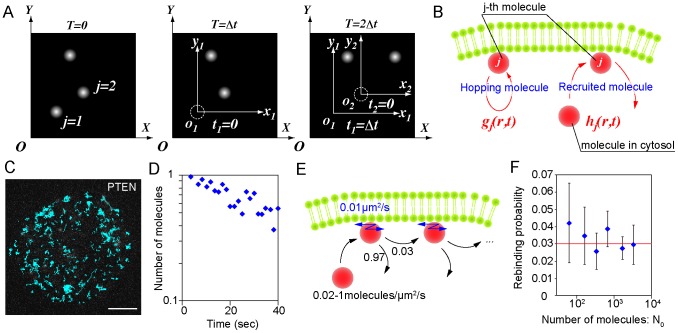
The analysis method for hopping molecules proposed in this study. (A) Two coordinate systems are introduced. In the global coordinate system, *O*(*X*,*Y*,*T*), the time origin is the initial frame and the spatial origin is the lower-left corner of the image. In the coordinate system about the *j*-th molecule, *o_j_*(*x_j_*,*y_j_*,*t_j_*), the time origin is the vanished time and the spatial origin is the vanished position of the molecule. (B) Membrane-associating molecules are classified into two types: those rebinding to the membrane after hopping (hopping molecule) and those recruited from the cytoplasm (recruited molecule). (C) The spatial distribution of wild-type PTEN trajectories (blue) observed in a single cell. Scale bar, 5 µm. (D) The number of molecules appeared in a single cell after excitation of the fluorophore. Time interval, 1.666 sec. (E) Schematics of the simulation of hopping molecules. (F) Results of the rebinding probability estimated from simulated molecules. Data are mean +/− SD.

From a movie of a single cell, we determined when and where every spot emerged and vanished by tracking the spot movement. A spot was regarded as vanished when there were no fluorescent spots in the next frame of the movie within *r*_0_ = 0.45 µm of the last position. The probability of a displacement larger than 0.45 µm, which is given by 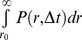, ranged from 0.002 to 0.05 (Table 4, Fig. S3). Based on this criterion, the trajectory of a hopping molecule, as shown in Fig. 3H, was separated into two. We introduced two coordinate systems to analyze the tracked data (Fig. 4A). One is a global coordinate system, *O*(*X*,*Y*,*T*), in which the spatial origin is the picture's corner and the time origin is the first frame of the movie. The other coordinate system varied with each spot, *o_j_*(*x_j_*,*y_j_*,*t_j_*), where *j* = 1,2,…, *N*_0_ (*N*_0_: total number of observed trajectories), *x_j_* and *y_j_* are spatial variables, and *t_j_* is a time variable. Note that the origin in this system is the vanished position and time of the *j*-th spot.

**Table 4:** Statistics of rebinding probabilities of wild-type PTEN and PTEN mutants.

Name	Mean	CI (upper)	CI (lower)	P value	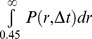
PTEN	0.086	0.107	0.064	-	0.00296
PTEN_1_	0.080	0.098	0.061	0.734	0.00353
PTEN_2_	0.067	0.094	0.039	0.340	0.00289
PTEN_3_	0.146	0.176	0.116	**0.007**	0.00737
PTEN_4_	0.155	0.220	0.090	**0.033**	0.01032
PTEN_5_	0.071	0.109	0.033	0.798	0.02349
PTEN_6_	0.043	0.091	−0.006	0.370	0.01835
PTEN_7_	0.017	0.129	0.044	**0.002**	0.01361
PTEN_4_+Latrunculin A	0.087	0.129	0.044	0.071	0.04239

The mean, 95% confidence interval and P value in two-sided *t*-tests are shown. P values are shown in bold when <0.05. The lateral diffusion probabilities showing displacements longer than 0.45 µm were calculated from the fitting of Eq. 2 to the data in Fig. S3.

First, we consider the situation when the *j*-th spot has vanished. The fluorescent spots that emerged near the vanished position potentially include both that derived from the hopping molecule and those derived from other molecules recruited from the cytosol (Fig. 4B). Thus, the probability density function (PDF) describing spot emergence at a distance *r* from the vanished position at time *t*, *f_j_*(*r*,*t*), is theoretically written as a summation of the PDF for spots that emerged by hopping, *g_j_*(*r*,*t*), and those that emerged by recruitment from the cytosol, *h_j_*(*r*,*t*), which leads to,
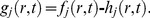
(3)
*f_j_*(*r*,*t*) is calculated by counting the number of spots which emerged in the area within a radius between *r* and *r*+Δ*r* and the time window between *t* and *t*+Δ*t*. The counted number was represented by the probability *f_j_*(*r*,*t*)2π*r*Δ*r*Δ*t*.

In order to model the PDF of the recruited molecules, *h_j_*(*r*,*t*), we assumed that the membrane association of cytosolic PTEN molecules occurs without any spatial heterogeneity on the membrane, an assumption validated by the homogeneous distribution of the molecular trajectories in wild-type cells (Fig. 4C). The number of spots that emerged between *T* and *T*+Δ*t* on the whole membrane was counted, divided by the area of the cell membrane, and plotted against time (Fig. 4D). The result shows a temporal decrease due to photo-bleaching and can be approximated by the exponential function,
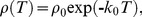
(4)where *ρ*_0_ and *k*_0_ are constants for the initial value and the decay rate, respectively. The PDF of the recruited molecules is expressed as,
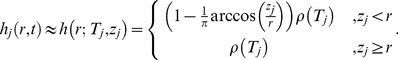
(5)
*z_j_* is the distance between the vanished position of the *j*-th molecule and the periphery of the cell. For the derivation, see *Materials and Methods*.

Next, in order to statistically analyze the PDF, a summation of Eq. 3 is taken as,
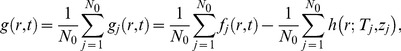
(6)where *N_0_* is the total number of molecules obtained directly by counting the spot number. According to the approximation that the cell size is much larger than *r*, Eq. 6 is re-expressed as
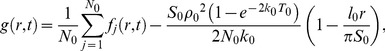
(7)where *l*_0_, *S*_0_ and *T*_0_ are the perimeter of the cell, the area of the cell membrane and the total time of the movie, respectively. Equation 7 is also valid for tracking data from *in vitro* single-molecule imaging assuming no effect of photo-bleaching and no limit in observation area, which gives rise to,
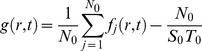
(8)(see *Materials and Methods* for details). Equation 8 coincides with Knight's model in which the rebinding probability is considered under *in vitro* imaging conditions [22].

To analyze the temporal change in the rebinding probability after a vanishing event, the probability between *t* and *t*+Δ*t* is calculated using a constant distance from the vanished position, *R*_0_, as,
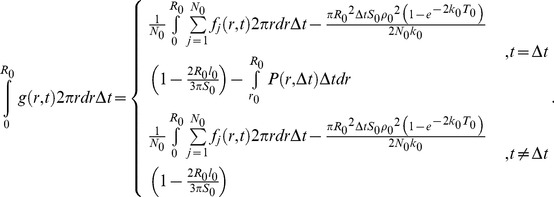
(9)When calculating the probability between Δ*t* and 2Δ*t*, the probability of lateral diffusion was subtracted, as shown in the third term on the right side. Hopping that occurred in the other time intervals can be clearly distinguished from lateral diffusion in the data collecting process, since two spots appeared in the same video frame (*t* = 0) or the spot once disappeared (*t*≥2Δ*t*). To analyze the spatial distribution of the rebinding probability around a vanished position, the probability within a radius *r* and *r*+Δ*r* was calculated using the constant *t* as,
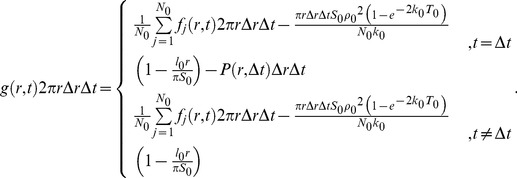
(10)A positive rebinding probability indicates that the membrane association frequency within *R*_0_ of the vanished position between *t* and *t*+Δ*t* after the vanishing time is higher than the average frequency of the membrane association; a rebinding probability around 0 indicates no significant differences between membrane association frequencies before and after the vanishing; and a negative rebinding probability indicates that a membrane association within *R*_0_ between *t* and *t*+Δ*t* is inhibited compared to the average frequency.

### Molecular number necessary for the rebinding probability estimation

In order to confirm our analysis method and examine the data size necessary to obtain confident estimations, we simulated molecular trajectories. We modeled molecules that exhibit hopping when they dissociate from the membrane assuming a rebinding probability of 0.03 (Fig. 4E), and the trajectories of the modeled molecules were generated by a stochastic simulation. The position and time each molecule emerged and vanished on the membrane were determined according to the same criterion as that used for measurements in living cells: when a molecule moved more than 0.45 µm in 33 msec, the trajectory before and after the displacement was regarded as derived from distinct molecules. Thus, the number of molecules, *N*_0_, recognized under this criterion in the analysis is generally larger than that in the simulations. Based on the recognized trajectories, the rebinding probability was calculated according to the proposed analysis method. For convenience, the probability that a molecule rebinds to the membrane within 2 µm of the vanished position and between 66 and 100 msec after the vanished time was used to confirm the analysis. Concerning each vanished molecule, the number of molecules that emerged within 2 µm of the vanished position and between 66 and 100 msec after the vanished time was counted. The counts obtained from all vanished molecules were summed, and the sum was divided by *N*_0_, providing the first term of the right side of Eq. 9. The number of molecules that emerged between *T* and *T*+Δ*t* sec of the simulation were counted irrespective of the position and fitted with Eq. 4 to provide estimates of *ρ*_0_ and *k*_0_. Using these estimated values as well as the values of the parameters *l*_0_, *N*_0_, *R*_0_, *S*_0_ and *T*_0_, the second term of the right side of Eq. 9 was calculated. The rebinding probability was estimated for each trial of the simulation, and 12 trials were performed to obtain an average and standard deviation of the estimated rebinding probabilities. Fig. 4F shows the relationship between the estimated rebinding probability and the number of analyzed molecules. The number of molecules analyzed in each trial was changed according to the membrane-association frequency (0.02–1 molecules/µm^2^/sec). The estimated value of the rebinding probability approached the simulated value, and the standard deviation decreased as the number of analyzed molecules increased. The results suggest that 1,000 molecules provide a good estimate for calculating the rebinding probability.

### Rebinding probability and hopping lifetime are regulated via the cα2 helix

**Figure 5:**
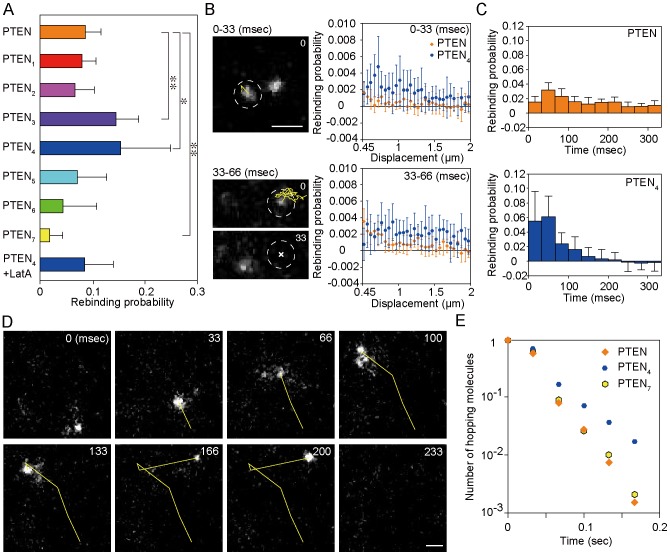
Rebinding probability analysis. (A) Rebinding probabilities of wild-type PTEN, PTEN mutants and Latrunculin A treated PTEN_4_ mutant. The probability that a molecule rebinds to the membrane between 0.45 and 2 µm from the vanished position and within 133 msec after the vanished time was calculated using Eq. 9. * and ** indicate p<0.05 and p<0. 01 obtained by Student's *t*-test, respectively. (B) Spatial distribution of rebinding probabilities of wild-type PTEN (orange) and PTEN_4_ (blue). The spatial distributions were obtained between 0 and 33 msec (upper) and 33 and 66 msec (lower) after spots vanished (right panels). The bin range is 0.05 µm. Typical images of hopping molecules that rebind between 0 and 33 msec (upper) and 33 and 66 msec (lower) are shown (left panels). The minimum limit of the analysis is 0.45 µm from the previous position (white dotted circles). Trajectories before hopping are shown in yellow. (C) Temporal changes in rebinding probability of wild-type PTEN (upper) and PTEN_4_ (lower). Data are mean +/− SD. (D) Typical images showing a succession of jumps and the corresponding trajectory (yellow). Numbers are time in milliseconds. Scale bar, 1 µm. (E) Hopping lifetimes of wild-type PTEN, PTEN_4_ and PTEN_7_.

The rebinding probabilities of PTEN in living *Dictyostelium* cells were estimated in the manner described above. The probability that a molecule rebinds to the membrane between 0.45 and 2 µm from the vanished position and within 133 msec after the vanished time was calculated in a single cell, and the probabilities obtained from more than 7 cells were averaged. Positive rebinding probabilities were obtained for all wild-type and mutant PTENs (Fig. 5A, Table 4). The rebinding probability of wild-type PTEN was estimated to be 0.08, indicating that rebinding takes place in 8% of membrane dissociations. The probabilities of PTEN_3_ and PTEN_4_ were twice as high as wild type, while those of the other PTEN mutants except for PTEN_7_ were similar to wild-type. Therefore, hopping is most likely to be an intrinsic molecular property of PTEN and suppressed when the cα2 helix has its intact state. The hopping probability did not exhibit direct correlation with the number of mutations in the cα2 helix, possibly due to a balance between association and dissociation rates, as discussed later.

Ranges of the displacement and time interval used for the rebinding probability estimation were chosen so that they included most hopping events based on analyses of the spatial distribution and temporal change in the rebinding probability. In the spatial distribution analysis, the displacement by hopping was examined using Eq. 10. The analysis was performed within a 33-msec time interval after the vanished time. The results obtained for the time intervals between 0 and 33 msec and 33 and 66 msec are representatively shown in Fig. 5B. When a hopped molecule rebound to the membrane within 0 and 33 msec, two fluorescent spots were observed in a single movie frame (Fig. 5B, upper-left). The rebinding probability was calculated in an area surrounded by two concentric circles with radii *r* and *r*+Δ*r* from the center of the pre-existing spot. The smallest possible radius in our analysis was *r* = 0.45 µm, which is shown as a dotted circle in the left panels of Fig. 5B. This limitation is due to the upper limit of the distance between two spots in neighboring frames, which are regarded as coming from the same molecule when generating the trajectories of lateral diffusion. The rebinding probability was calculated assuming Δ*r* = 0.05 µm and was maximal at around *r* = 0.45 µm, but decreased to 0 as *r* approached 2 µm (orange in the upper-right panel of Fig. 5B). Similar spatial distributions were obtained from the analyses in the time interval between 33 and 66 msec (orange in the lower-right panel of Fig. 5B). Therefore, wild-type PTEN molecules hopped at most 2 µm on the membrane.

Temporal changes in the rebinding probability were analyzed using Eq. 9. A sum of the probabilities from *r* = 0.45 to 2 µm in the spatial distribution was taken to calculate the probability that a molecule rebounds to the membrane during an arbitrary time interval. As a consequence, the rebinding probability of wild-type PTEN was highest between 33 and 66 msec after the time the spot vanished (Fig. 5C, upper panel). Because the majority of rebinding events occurred before 133 msec, we chose the time interval 0–133 msec when calculating the rebinding probabilities shown in Fig. 5A.

These spatial and temporal characteristics were largely shared among all PTEN mutants except PTEN_4_ (Figs. 5B, 5C, S4 and S5). The PTEN_4_ rebinding probability showed broader distributions with higher values than that of wild type, demonstrating that it retains rebinding ability even after travelling long distances (blue in right panels of Fig. 5B). The timing of rebinding was also faster compared to wild-type and other mutant PTENs (Fig. 5C, S5). Thus, PTEN_4_ is more likely to rebind to the membrane within 0.45 and 2 µm of the vanished position and within 0 and 133 msec after the vanished time than wild-type.

We further confirmed that the cα2 helix is essential for the suppression of hopping by measuring the time duration of a succession of hops. As shown in Fig. 5D and the supplemental movie S2, we sometimes observed hopping for several frames, especially in the case of PTEN_4_. To analyze the time duration of the hopping, which we named “hopping lifetime,” we regarded two spots that emerged within 1 frame as from the same molecule when the spatial and temporal distances between the spots were within 2.0 µm and 33 msec. The trajectory of the succession of hopping was reconstructed (yellow lines in Fig. 5D), and the time length was measured. In the cumulative plots of the hopping lifetimes, PTEN_4_ showed a longer hopping lifetime than wild-type PTEN or PTEN_7_ (Fig. 5E). The three plots were fitted to a single exponential function, *A*exp(-*kt*), giving rise to a rate constant in which PTEN_4_ had the lowest value (Table 5). Together with the result indicating PTEN_4_ has a higher rebinding probability than wild type or PTEN_7_ (Fig. 5A), we concluded that PTEN can adopt a specific state in which hopping occurs at a high frequency.

**Table 5:** Single exponential fitting parameters for hopping lifetimes in Fig. 6C.

Name	*A*	*k* [1/sec]
PTEN	2.7	42.0
PTEN_4_	1.1	23.5
PTEN_7_	2.5	40.0

**Figure 6:**
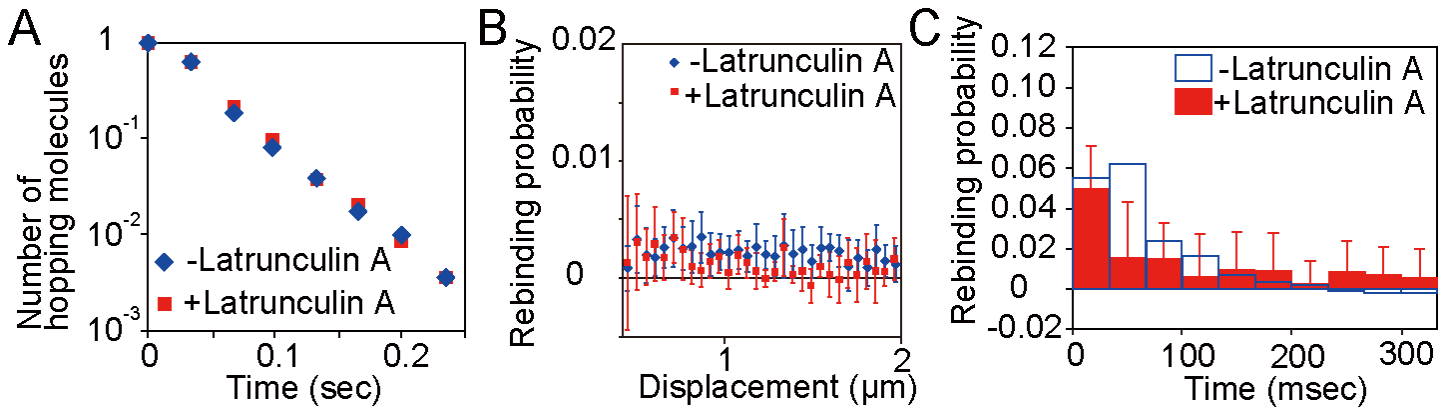
Effect of actin filaments on PTEN hopping. (A) Hopping lifetimes of PTEN_4_ in the absence (blue) and presence (red) of Latrunculin A. (B) Spatial distribution of the rebinding probability of PTEN_4_ in the absence (blue) and presence (red) of Latrunculin A. (C) Temporal changes in the rebinding probability of PTEN_4_ in the absence (blue) and presence (red) of Latrunculin A. Data are mean +/− SD.

Consistent with this idea, the state of actin polymerization beneath the plasma membrane hardly affected the hopping lifetime (Fig. 6A). Treatment with an actin polymerization inhibitor, Latrunculin A, did not change the hopping lifetime of PTEN_4_, whereas it decreased the rebinding probability at short time intervals and long distances from the vanishing time and position, respectively (Fig. 5A, 6B and C). These results indicate that PTEN intrinsically adopts the hopping state and that the rebinding probability is spatiotemporally regulated by the density of cortical filamentous actin.

**Figure 7:**
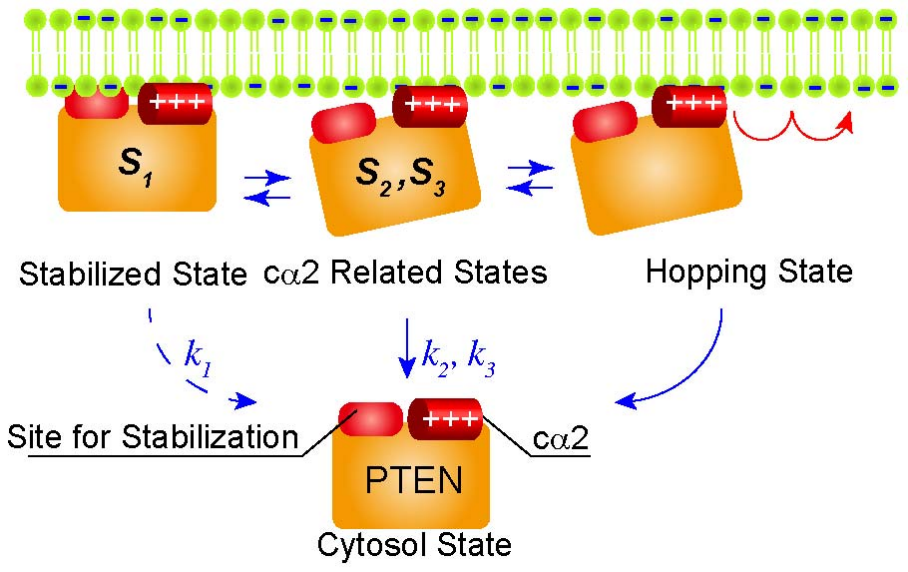
A “search-and-stabilization” model describes the role of the cα2 helix in the PTEN-membrane interaction. PTEN mainly adopts three states on the membrane: a stabilized state (*S*_1_), two cα2-related states (*S*_2_, *S*_3_) plus a hopping state. The cα2 helix is involved in suppressing the membrane dissociation of PTEN by directly regulating the latter two states. When the state changes from *S*_2_ or *S*_3_ to *S*_1_, the membrane interaction becomes stabilized and the substrate PI(3,4,5)P_3_ is more accessible. In the *S*_2_ and *S*_3_ states, PTEN exhibits membrane dissociation at a faster rate than in the *S*_1_ state. Of the dissociating molecules, 8% rebind to the membrane after hopping, which offers a chance for PTEN to search for the substrate again.

Finally, the contribution of the cα2 helix on the membrane interaction is summarized (Fig. 7). Three states, *S*_1_ (*S′*_1_), *S*_2_ (*S′*_2_) and *S*_3_ (*S′*_3_), were revealed from the analysis of the membrane dissociation kinetics and lateral diffusion mobility (Fig. 2, Table 2 and 3). The mutation suppressed *S*_1_ but enhanced *S*_2_ and *S*_3_, indicating that positive charges are involved in the state transition between *S*_1_, the stabilized state, and *S*_2_ and *S*_3_, cα2-related states. In addition to these three, we found that PTEN adopts a hopping state, which may indicate a specific conformation where PTEN is unusually sensitive to electrostatic interactions with the membrane (Fig. 5, 6). The PTEN_4_ mutation most likely accelerates dissociation from the membrane in the *S*_2_ and *S*_3_ states and induces the hopping state. Once dissociated from the membrane, 8% of wild-type PTEN molecules rebind to the membrane near their original location without diffusing into the cytoplasm. Because the intracellular PTEN distribution is almost constant over time, all membrane-associating molecules should include those bound to the membrane by hopping. Based on these results, we propose two types of membrane association reactions for PTEN: association by diffusing from the cytoplasm and association by hopping on the membrane surface. The cα2 helix, which locates on the interface of the membrane interaction, is responsible for electrostatic membrane bindings and most likely central to the regulation of the amount of PTEN on the membrane by modulating the likelihood of hopping.

## Discussion

In this study, we demonstrated that localization of PTEN to the cell membrane is regulated via positively charged residues in the cα2 helix of the C2 domain, which is indispensable for proper PTEN function in phosphatidylinositol metabolism and *Dictyostelium* developmental progression. The localization shift from the membrane to cytosol was observed after decreasing the positive charge of the cα2 helix, which partly destabilized the membrane interaction, as revealed quantitatively by single-molecule imaging (Fig. 1, 2). In some circumstances, single PTEN molecules were seen to undergo large lateral diffusion on the cell membrane, appearing as though they hopped extraordinarily large distances (Fig. 3). A novel statistic analysis method was developed to estimate the probability that a molecule rebinds to the membrane soon after it dissociates (Fig. 4). The estimation for all wild-type and mutant PTEN molecules demonstrated that the positive charge of the cα2 helix was essential for suppressing hopping (Figs. 5, 6). Based on these results, we proposed a model describing multiple membrane-binding states of PTEN in which the cα2 helix are required for PTEN accumulation on the membrane by strengthening the electrostatic interactions in three relatively stable membrane-binding states, *S*_1_, *S*_2_ and *S*_3_, and suppressing state transitions into the unstable hopping state (Fig. 7).

We demonstrated that the cα2 helix is necessary for the localization of PTEN to the cell membrane in living cells. The membrane localization depended on the number of amino-acid substitutions in the cα2 helix, suggesting the involvement of electrostatic interactions. A previous crystallography study showed that PTEN contains two cationic patches on the interface of the membrane interaction that are derived from positively charged residues in the cα2 helix and CBR3 loop in the C2 domain [8]. That report concluded the electrostatic interactions between these cationic patches and anionic phospholipids such as PI(4,5)P_2_ and PS are indispensable for phospholipid phosphatase activity on the cell membrane. However, until the present report, no suggested mechanism of the recruitment of PTEN to the membrane in living cells has involved the cα2 helix (Fig. 1).

Moreover, it has been suggested that the CBR3 loop of mammalian PTEN is masked by a multi-phosphorylated C-terminal tail [13]. Therefore, the CBR3 loop and C-terminal tail together may provide an additive regulatory module for the membrane interaction. However, we could not find any amino-acid stretches homologous to the CBR3 loop or C-terminal tail in *Dictyostelium* PTEN. In addition, membrane localization of *Dictyostelium* PTEN is also dependent on the N-terminal PI(4,5)P_2_-binding motif, suggesting that electrostatic interactions via the cα2 helix and PI(4,5)P_2_-binding motif are primarily responsible for recruiting PTEN to the cell membrane in living cells [9]. The relevance of these cationic patches to *in situ* tumor-suppressing activity should be explored, since tumor-derived mutations were found more frequently in the cα2 helix than the CBR3 loop (see the Catalogue of Somatic Mutations in Cancer (COSMIC) website) [8], [25].

We revealed that the positively charged residues in the cα2 helix stabilize membrane interactions of PTEN. PTEN exhibited membrane dissociation at three rates, indicating that it adopts at least three kinetic states (Fig. 2B, Table 2). Similarly, PTEN showed three diffusion coefficients during the membrane binding, indicating three diffusion states. We have previously reported that the phosphatase-minus form of PTEN (PTEN_G129E_) adopts three states: one with the slowest rate and lowest mobility, one with the fastest rate and highest mobility, and the third with a moderate rate and mobility [26]. The three states most likely arise due to different membrane interaction stabilities. In this study, we found that the frequency of adopting the slowest rate and the lowest mobility state (*S*_1_) decreased with increasing neutral charge, while the other two states (*S*_2_ and *S*_3_) increased. Thus, we concluded that the cα2 helix possibly modulates state transitions (Fig. 7). The positively charged residues are necessary for enhancing *S*_1_, which is stable, and suppressing the other two, *S*_2_ and *S*_3_, which are relatively unstable. Consequently, membrane dissociation is suppressed due to the positive charge, which causes an accumulation of PTEN on the membrane. However, because *S*_1_ is more strongly bound to the membrane than *S*_2_ and *S*_3_, it likely requires other interaction sites on the membranes besides the anionic phospholipids recognized by the cα2 helix. Further studies should investigate which molecules on the membrane associate with PTEN in these states and the PTEN structures to clarify the mechanism of the membrane interactions and state transitions.

Along with the above three states, single molecule imaging revealed a fourth state that describes PTEN hopping on the membrane of living *Dictyostelium* cells. These hopping molecules showed extraordinary large displacements, typically 0.5 µm per 33 msec, compared to the 0.08 µm per 33 msec seen by diffusing molecules (Fig. 3). Such a phenomenon has been reported only once before, but never in living cells [22]. Because our *in vivo* observation of hopping is unprecedented, we required a novel analysis method to confirm that the fluorescence signals before and after hopping were from the same molecule. We estimated the probability that a molecule once dissociated from the membrane instantly rebinds by analyzing video images of single molecules *in vivo*, which are different from images of molecules *in vitro* in the following two points (Fig. 4). First, the volume of the cell is smaller than that of a typical reconstituted system and thus contains fewer fluorescently labeled molecules. Second, the size of the cell in which the association, dissociation and hopping take place is smaller than that of the image (Fig. 2A). By assuming infinitely slow photo-bleaching and an infinitely large cell boundary, our *in vivo* rebinding probability approximated the *in vitro* probability [22]. In addition, we could estimate the probability of where and when rebinding occurred relative to the vanished position and time (Figs. 5B, 5C, S4, S5). Therefore, our analysis method for hopping molecules generally applies to both *in vitro* and *in vivo* single-molecule imaging data and provides detailed spatio-temporal properties of hopping.

Statistical analyses of the rebinding probability of all wild-type and mutant PTENs revealed that the cα2 helix is involved in the suppression of hopping. The probability that wild-type PTEN rebinds to the membrane within 2 µm and 133 msec of the vanished position and time was estimated to be 0.08 in living *Dictyostelium* cells (Fig. 5A), and most rebinding events occurred within these spatio-temporal limits (Figs. S2 and S3). Therefore, 8% of dissociated PTEN molecules rebound to the membrane after hopping and 92% diffused into the cytoplasm. The rebinding probability was highest for PTEN_4_, in which charge-decreasing substitutions were introduced moderately (Fig. 5A). The rebinding probability is thought to maximize when the dissociation rate constant equals the association rate constant multiplied by the concentration of the molecule in the solution [27]. When the dissociation rate constant is close to zero, binding sites are occupied so that rebinding hardly occurs, and when it approaches infinity, the membrane works as a reflecting wall. In either case, there is no rebinding. On the other hand, moderate dissociation rate constants cause a high frequency of rebinding. Another explanation for maximum rebinding probability might be due to a structural change in the cα2 helix by the mutation. Single molecule imaging of PTEN_4_ at several concentrations on supported lipid bilayers should clarify the cause.

We also sometimes observed a succession of hops by PTEN_4_ for several movie frames (Fig. 5D). Successive hopping may represent a specific state in which PTEN is unusually adsorptive to the membrane. Consistent with this premise, the lifetime of successive hopping obeyed an exponential distribution and was unaffected by treatment with an actin polymerization inhibitor, Latrunculin A (Fig. 5E, Table 5). Therefore, we concluded that PTEN most likely adopts a metastable state in which hopping dominates binding to the membrane (Fig. 7). The state of the actin cytoskeleton, including possibly the density of the cortical meshwork, affected the rebinding probability, which was reduced upon Latrunculin A treatment (Fig. 6). These results suggest that PTEN intrinsically exhibits hopping but rebinds in a manner dependent on the environmental conditions beneath the membrane.

Based on above, we propose a model describing the role of the cα2 helix in the membrane interaction of PTEN. PTEN mainly adopts four states: a stabilized state (*S*_1_), two cα2-related states (*S*_2_, *S*_3_) and a hopping state. *S*_1_, *S*_2_ and *S*_3_ are membrane-bound states with *S*_1_ being more stable than *S*_2_ or *S*_3_. In the hopping state, PTEN is not bound to the membrane but adsorbed nearby by electrostatic interactions, thus it occasionally shows repetitive hopping on the membrane. The *S*_2_, *S*_3_ and hopping states directly depend on the integrity of the cα2 helix, which maintains the *S*_2_ and *S*_3_ states but suppresses the hopping state. The hopping state could serve as an electrostatic search on the membrane for PTEN to find its substrate, as suggested previously [22]. In our model, interactions between PTEN and PI(3,4,5)P_3_ are thought to be enhanced by a “search and stabilization” mechanism. PTEN in the *S*_2_ and *S*_3_ states is weakly bound to anionic phospholipids like PS via the C2 domain due to the surface positive charge of the cα2 helix. When membrane binding is stabilized through interactions via both the C2 and phosphatase domains, PTEN takes the *S*_1_ state, which makes it accessible to PI(3,4,5)P_3_ and leads to activation of the phosphatase. Otherwise, PTEN adopts the hopping state at a non-negligible rate. The majority of hopping PTEN molecules then diffuses into the cytoplasm, but some (8%) rebinds to the membrane instead. Thus, PTEN can escape a local region with relatively low substrate concentrations by hopping. Although whether the transitions between states are modulated by local phospholipid compositions or the electrostatic state of the local membrane requires further investigation, we suspect that the cα2 helix works as a sensor for the membrane microenvironment. Thus, despite PI(3,4,5)P_3_ being a rare component of membrane phospholipids, the search and stabilization mechanism provides an efficient way for PTEN to encounter and dephosphorylate its substrate, which can be critical not only for the metabolism of the signaling phospholipid in single cells but also for the size regulation and progression of multicellular development.

## Materials and Methods

### Plasmid construction

Plasmids for the extrachromosomal expression of PTEN*_i_*-Halo were constructed based on the same vector as previously reported [26]. Mutations for basic amino acids substitutions in the cα2 helix were introduced into the *pten* gene cloned into pCR4BluntTOPO (Invitrogen) according to the manufacturer's manual (QuikChange II XL Site-Directed Mutagenesis Kit, Agilent Technologies). The primers used are shown in Table 1. The resultant nucleotide sequences were confirmed by sequencing. The mutant genes were cloned into the *Bgl*II and *Spe*I sites of pHK12neo to generate in-frame fusion with the gene encoding HaloTag between the promoter and terminator sequences. 5 µg of the expression plasmids were used for the transformation. Transformation of the plasmids for the expression of PTEN-Halo or PTEN*_i_*-Halo was performed as described previously [26]. The transformed cells were selected under 10 µg/ml of G418 for 1 week.

### Cells preparation

*Dictyostelium discoideum* wild-type cells (Ax2) were grown at 21°C in HL5 medium supplemented with 100 ng/ml folic acid and 5 ng/ml vitamin B12. Transformation of the plasmids for the expression of PTEN-Halo or PTEN*_i_*-Halo was performed as described previously [28]. The transformed cells were selected under 10 µg/ml of G418 for 1 week. Before microscopic observations, cells were starved in 1 ml developmental buffer (DB; 5 mM Na/KPO_4_, 2 mM MgSO_4_, 0.2 mM CaCl_2_, pH6) at a density of 2×10^6^ cells/ml for 1 hour and then incubated for another 3 hours in the presence of 10 µl pulses of 1 µM cAMP given every 6 minutes. For the observation of fruiting bodies, cells were washed with DB, and 20 µl of the cell suspension at a density of 4×10^7^ cells/ml was plated on 1.5% non-nutrient agar for 30 hours.

### Confocal imaging

Prepared cells were incubated in DB including 10 µM TMR conjugated HaloTag ligands (Promega) for 10 minutes. The cells were washed by DB, incubated on a cover glass for 10 min and observed by a confocal microscope (TE2000-PFS, Nikon). PTEN-TMR was excited by 561-nm solid state CW lasers. The average intensities on the plasma membrane and in the cytoplasm were calculated using Image-Pro (Media Cybernetics).

### Single molecule imaging

To label wild type PTEN-Halo or PTEN*_i_*-Halo, prepared cells were incubated with DB including 100 nM TMR conjugated HaloTag ligands for 10 minutes. The cells were then washed and suspended in DB and placed on a glass cover slip. After 10 minutes, cells were overlaid with a sheet of agarose and incubated for 20 minutes [29]. To inhibit actin polymerization, cells were treated with 10 µM Latrunculin A (Invitrogen). The sheet of agarose was also treated with 10 µM Latrunculin A before use. Single molecules of wild type PTEN-Halo and PTEN*_i_*-Halo were visualized through objective type TIRFM. Details of the TIRFM configuration are described elsewhere [23].

### Single-particle tracking

The trajectories of fluorescent spots were obtained automatically from the movies using laboratory-made software and involved three steps: determine the position of the fluorescent spots observed in each frame of the movie, acquire the trajectories of the movement by temporally connecting the position data, and eliminate artifactual trajectories that arose due to noise in the images. To determine the position, a two-dimensional fluorescence intensity profile for each spot was fitted to a Gaussian function after the frame image was binarized. The binarization was performed using the correlation coefficients of the intensity calculated from neighboring pixels. The precise position was determined in the binarized image by fitting the Gaussian function, *I*exp(−((*x*-*x*_0_)^2^+(*y*-*y*_0_)^2^)/2*σ* ^2^)+*J*, where *I*, *x*_0_, *y*_0_, *σ*, and *J* are the peak intensity, *x* position, *y* position, deviation, and background fluorescence intensity, respectively. To obtain the trajectories, the spots observed in one frame and the previous frame were connected if the displacement was smaller than a threshold, *d* = 0.45 µm. If there were multiple spots in the connection region, the nearest one was selected. To distinguish artifactual trajectories derived from background noise, a signal to noise ratio (SNR), 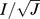, and *σ* were calculated at each time point along the trajectory of an individual spot [30]. The trajectories with an average SNR not more than 5 or an average deviation not between 1 and 4 were regarded as artifacts and removed from the analysis.

### Derivation of rebinding probability

The PDF for the recruited molecules is expressed as,
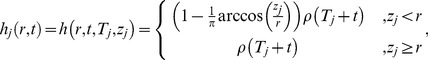
(11)where *z_j_* is the distance between the vanished position of the *j*-th molecule and the periphery of the cell. If *z_j_* is smaller than *r*, the circle of radius *r* protrudes from the cell, and the length of the protruded arc is 2*r*arccos(*z_j_*/*r*). The ratio of the length to the circumference, 2π*r*, is arccos(*z_j_*/*r*)/π. Equation 11 excludes the protruded length. In addition, *ρ*(*T_j_+t*) includes not only the molecules recruited from the cytosol but also the *j*-th molecule, whereas *h_j_*(*r*,*t*) only includes the former. However, since the total number of molecules observed in the movie was much larger than that of the *j*-th molecules, we concluded that *h_j_*(*r*,*t*) approximates to *ρ*(*T_j_+t*). Furthermore, since we only consider the situation with small *t*, the value of *ρ*(*T+t*) can be approximated as *ρ*(*T*). Therefore, Eq. 11 can be rewritten as Eq.5.

In obtaining a summation of Eq. 3, the second term on the right side of Eq. 6 is written as,
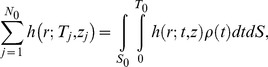
(12)since the distribution of *T_j_* depends on *ρ*. If the cell size is much larger than *r*, we have the approximation:
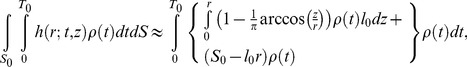
(13)where *l*_0_ is the perimeter of the cell. Using Eqs. 12 and 13, Eq. 6 is re-expressed as Eq. 7.

Equation 7 is also valid for tracking data from *in vitro* single-molecule imaging. In typical cases, a volume of the solution in which fluorescent molecules diffuse is much larger than a cell's volume and the effect of photo-bleaching is insignificant. Since the total number of molecules is calculated as,
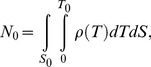
(14)where *S*_0_ and *T*_0_ are the area of the cell membrane and the total time of the movie, respectively, *ρ*(*T*) approximates to a constant value and we obtain:
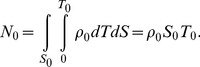
(15)From Eq. 15, Eq. 7 is rewritten as,
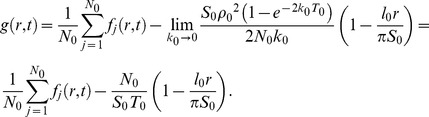
(16)In addition, the observation area is not limited to the cell. *S*_0_ approaches infinity, so *l*_0_/*S*_0_ can be approximated to 0, which gives rise to Eq. 8.

### Simulation for hopping molecules

To estimate a sufficient number of trajectories for calculating the rebinding probability, we performed stochastic simulations to generate molecular trajectories followed by the analysis proposed in this study. The simulation was based on a model in which molecules show membrane associations and dissociations that include lateral diffusion and hopping and also incorporated the photo-bleaching of fluorophores. The molecular behaviors assumed in the model were as follows. A molecule in the cytoplasm associates with the membrane at a frequency of 0.02–1 molecules/µm^2^/sec. After association, the molecule exhibits lateral diffusion with a diffusion coefficient of 0.01 µm^2^/sec and dissociates from the membrane at a rate constant of 1/sec. The dissociated molecule has a chance to jump and rebind to the membrane at a rebinding probability of 0.03. The hopping molecule has a diffusion coefficient in the cytoplasm of 1 µm^2^/sec and returns to the membrane 66 msec after its dissociation. After returning to the membrane, the molecule shows the same behavior as before hopping. Irrespective of the molecular state, the fluorophore conjugated to the molecule undergoes photo-bleaching at a rate constant of 0.03/sec, which limits the trajectory length. The simulation was performed on a 10×10 µm membrane with a time interval of 33 msec and duration of 100 sec (3000 steps). To simulate the displacement of the diffusing molecules, we generated a normal random number whose standard deviation was 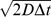 to define the displacement of molecules diffusing on the membrane. To differentiate the number of observed molecules (Fig. 4D), the membrane association frequency was changed. The order of the parameters used in the simulation was similar to that obtained from the single molecule imaging of PTEN *in vivo*.

## Supporting Information

Figure S1 Fluorescence intensities of wild-type PTEN and PTEN mutants in single-molecule imaging. (A) Histograms of fluorescence intensities. (B) Single-step photo-bleaching. The histogram has one peak and the fluorescence intensity suddenly drops, which indicates that the observed spots are single molecules.
(TIF)

Figure S2 Dissociation curves. The experimental data seen in Fig. 2B (dots) were fitted to two- (dotted lines) and three- (lines) component exponential function using Eq. 1 by the least squares method. The parameters for the two-component fit are shown in Table S1.
(TIF)

Figure S3 Displacement distribution analysis. The displacement distributions of wild-type and mutant PTEN molecules measured during 33 msec (red) were fitted to two- (dotted lines) and three- (blue) component diffusion probability functions using Eq. 2 by the least squares method. The bin range is 0.001 µm. The parameters for the fits are shown in Tables S2 and 3.
(TIF)

Figure S4 Spatial distribution of rebinding probabilities of wild-type PTEN and PTEN mutants. Colorless rectangles show the rebinding probability before subtraction of the lateral diffusion probability. The bin range is 0.05 µm. Data are mean +/− SD.
(TIF)

Figure S5 Temporal distribution of rebinding probabilities of wild-type PTEN and PTEN mutants. Colorless rectangles show the rebinding probability before subtraction of the lateral diffusion probability. Data are mean +/− SD.
(TIF)

Movie S1 Single-molecule imaging of wild-type PTEN and PTEN mutants in *Dictyostelium discoideum* cells as seen in Fig. 2. Scale bar, 5 µm. Frame rate, 30 fps.
(MP4)

Movie S2 Single-molecule imaging of PTEN_4_ hopping in *Dictyostelium discoideum* cells as seen in Fig. 3. The molecule undergoing lateral diffusion (left) and the molecule undergoing repetitive hopping (right, labeled in red) are shown with their trajectories (yellow). Scale bar, 1 µm. Frame rate, 5 fps.
(MP4)

Table S1 Dissociation rate constants of wild-type PTEN and PTEN mutants in the two-component model. Parameters obtained from fitting the data in Fig. 2B with Eq. 1 (*n* = 2). See also Fig. S2.
(DOCX)

Table S2 Diffusion coefficients of wild-type PTEN and PTEN mutants in the two-component model. Parameters obtained from fitting the data in Fig. S3 with Eq. 2 (*n* = 2). The observable lowest *D* was *ε* ^2^/Δ*t*, where *ε* is a standard deviation of measurement error calculated from the mean square displacement of the trajectories [20].
(DOCX)
