# Supplementary figures and images for: PTEN Hopping on the Cell Membrane Is Regulated via a Positively-Charged C2 Domain
Source: PLoS Comput Biol. 2014 Sep 11;10(9):e1003817. doi: 10.1371/journal.pcbi.1003817 (PMC4161299; doi:10.1371/journal.pcbi.1003817)

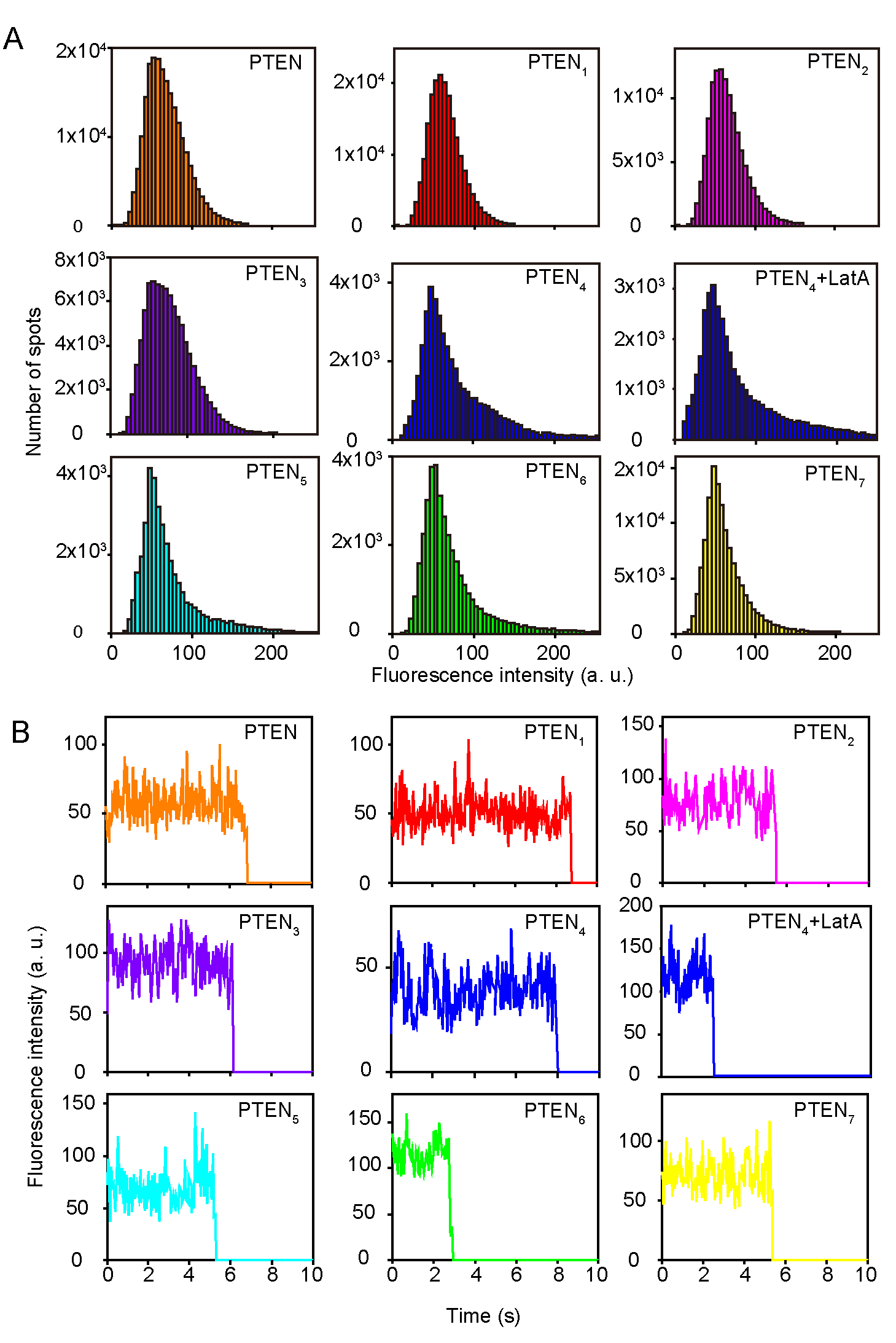

Supplement: Figure S1 — Fluorescence intensities of wild-type PTEN and PTEN mutants in single-molecule imaging. (A) Histograms of fluorescence intensities. (B) Single-step photo-bleaching. The histogram has one peak and the fluorescence intensity suddenly drops, which indicates that the observed spots are single molecules. (TIF) [file pcbi.1003817.s001.tif]

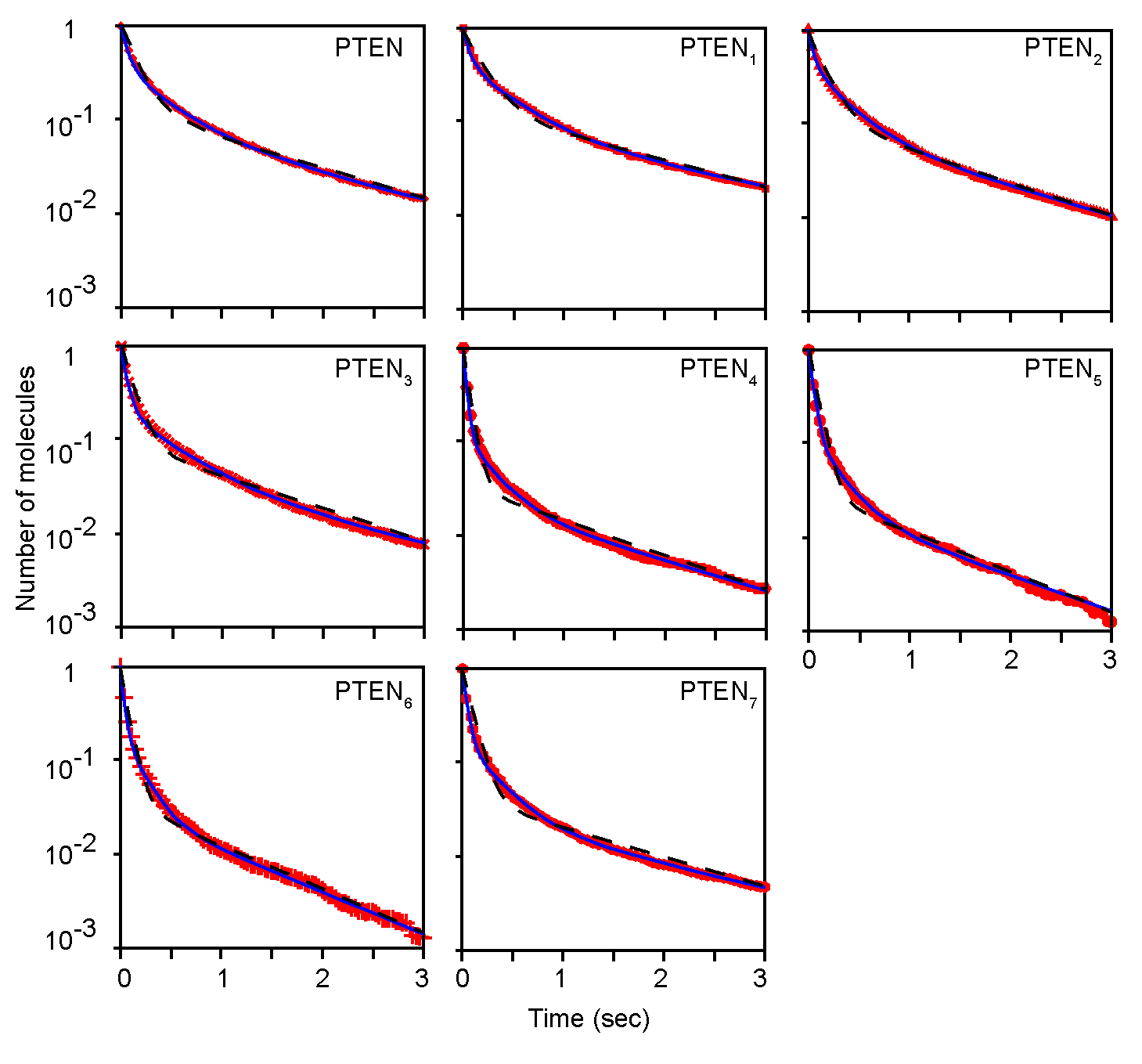

Supplement: Figure S2 — Dissociation curves. The experimental data seen in Fig. 2B (dots) were fitted to two- (dotted lines) and three- (lines) component exponential function using Eq. 1 by the least squares method. The parameters for the two-component fit are shown in Table S1. (TIF) [file pcbi.1003817.s002.tif]

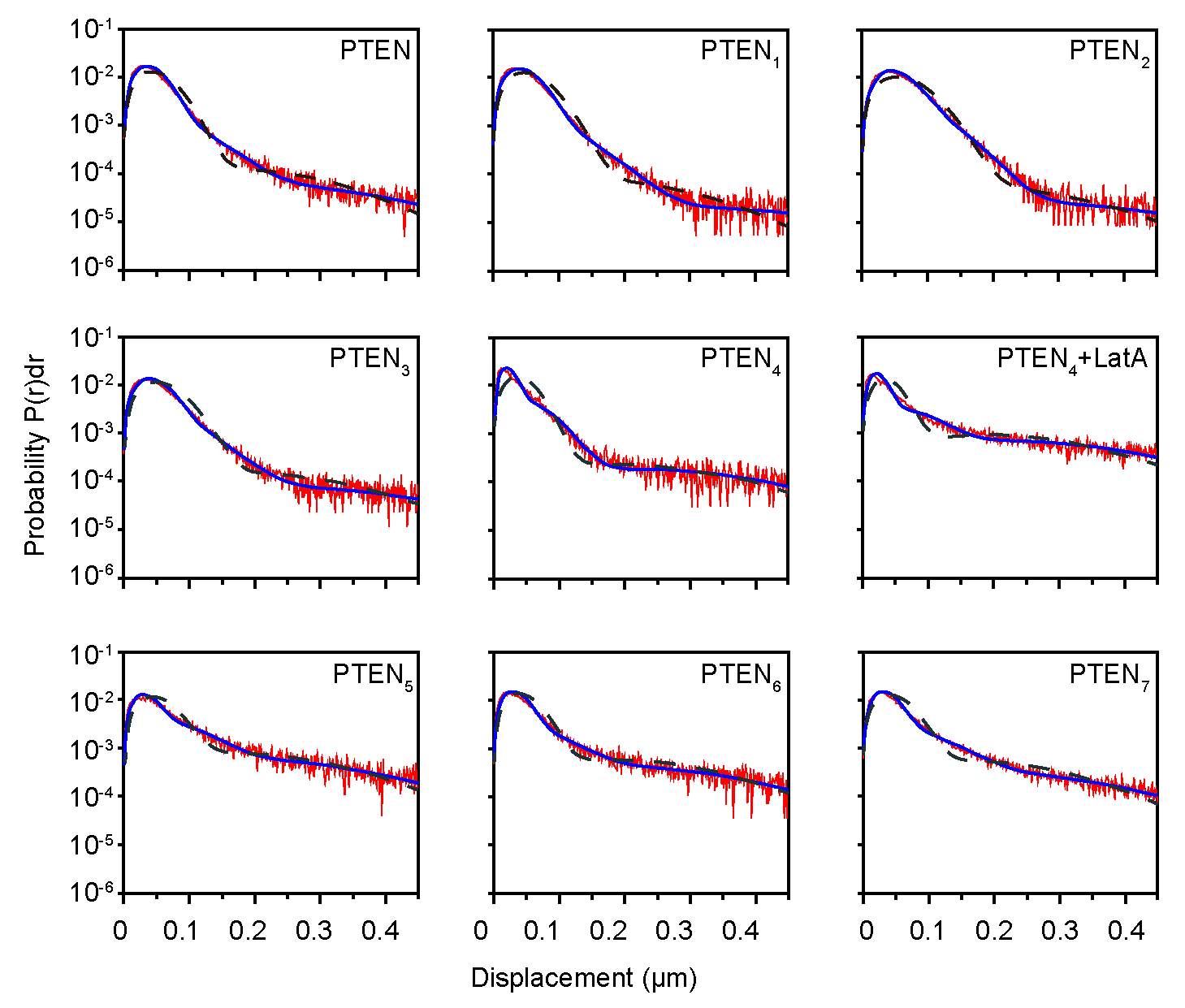

Supplement: Figure S3 — Displacement distribution analysis. The displacement distributions of wild-type and mutant PTEN molecules measured during 33 msec (red) were fitted to two- (dotted lines) and three- (blue) component diffusion probability functions using Eq. 2 by the least squares method. The bin range is 0.001 µm. The parameters for the fits are shown in Tables S2 and 3. (TIF) [file pcbi.1003817.s003.tif]

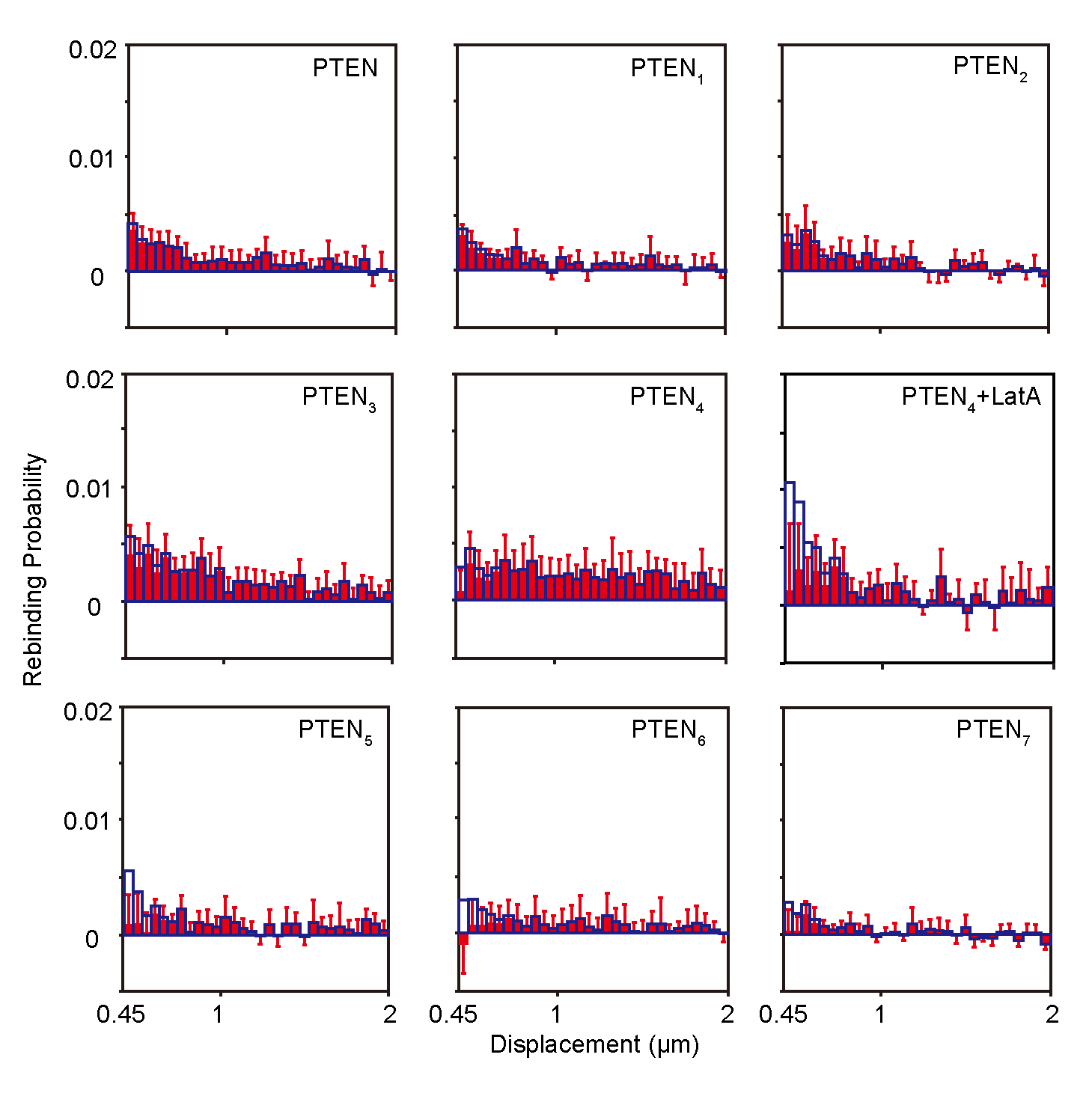

Supplement: Figure S4 — Spatial distribution of rebinding probabilities of wild-type PTEN and PTEN mutants. Colorless rectangles show the rebinding probability before subtraction of the lateral diffusion probability. The bin range is 0.05 µm. Data are mean +/− SD. (TIF) [file pcbi.1003817.s004.tif]

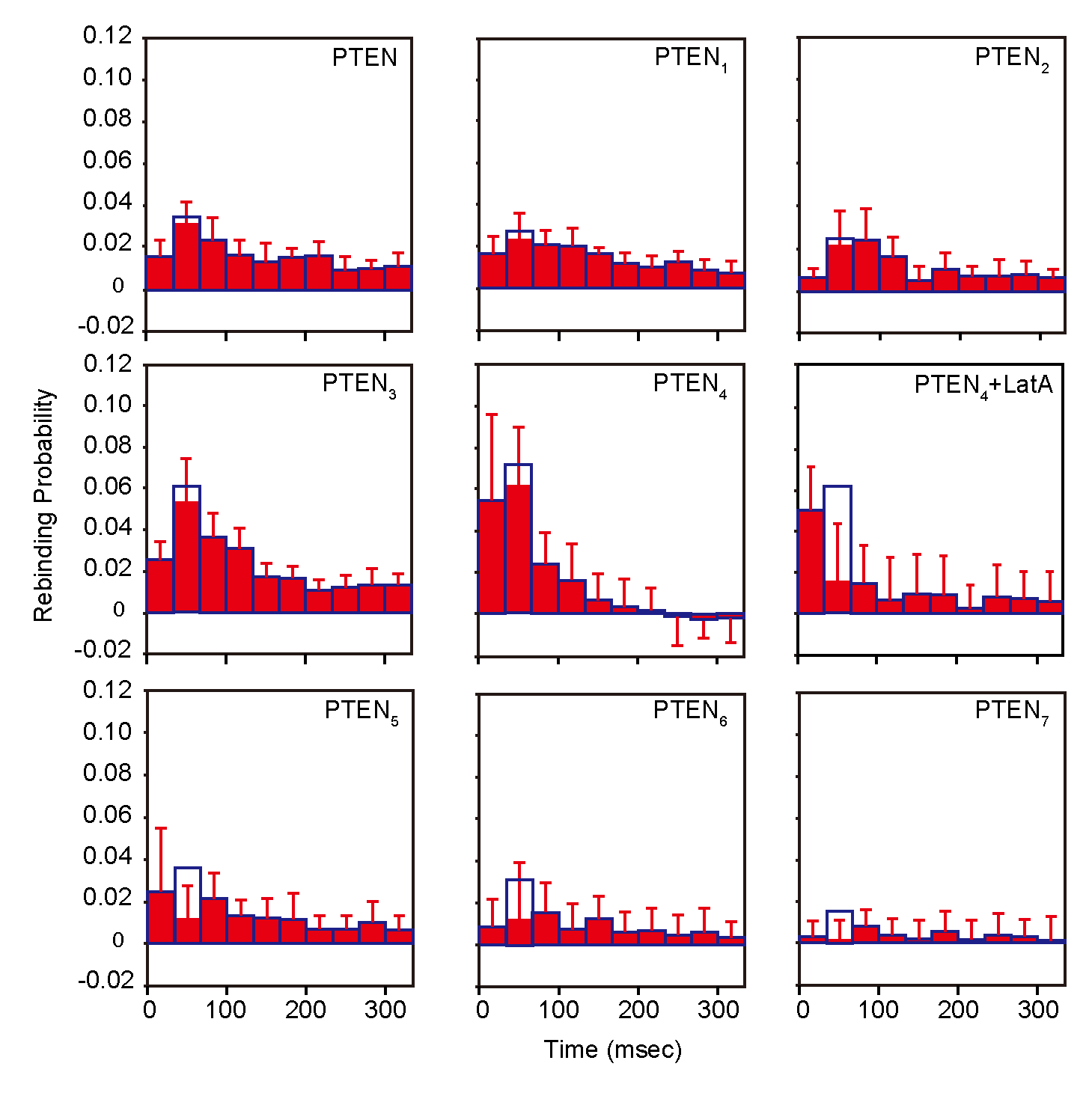

Supplement: Figure S5 — Temporal distribution of rebinding probabilities of wild-type PTEN and PTEN mutants. Colorless rectangles show the rebinding probability before subtraction of the lateral diffusion probability. Data are mean +/− SD. (TIF) [file pcbi.1003817.s005.tif]
