# Supplementary material for: PTEN Hopping on the Cell Membrane Is Regulated via a Positively-Charged C2 Domain
Source: PLoS Comput Biol. 2014 Sep 11;10(9):e1003817. doi: 10.1371/journal.pcbi.1003817 (PMC4161299; doi:10.1371/journal.pcbi.1003817)
Supplement: Table S1 — Dissociation rate constants of wild-type PTEN and PTEN mutants in the two-component model. Parameters obtained from fitting the data in Fig. 2B with Eq. 1 (n = 2). See also Fig. S2. (DOCX) [file pcbi.1003817.s008.docx]

**Table S1**. Dissociation rate constants of wild-type PTEN and PTEN mutants in the two-component model.

| ***NAME*** | ***A*_1_** | ***A*_2_** | ***k*_1_ [1/sec]** | ***k*_2_ [1/sec]** |
| --- | --- | --- | --- | --- |
| PTEN | 0.136 | 0.864 | 0.74 | 6.38 |
| PTEN_1_ | 0.141 | 0.859 | 0.64 | 5.38 |
| PTEN_2_ | 0.121 | 0.879 | 0.81 | 6.37 |
| PTEN_3_ | 0.090 | 0.910 | 0.80 | 9.24 |
| PTEN_4_ | 0.034 | 0.966 | 0.86 | 17.09 |
| PTEN_5_ | 0.031 | 0.969 | 0.97 | 13.19 |
| PTEN_6_ | 0.037 | 0.963 | 1.08 | 13.39 |
| PTEN_7_ | 0.041 | 0.959 | 0.71 | 10.64 |

Parameters obtained from fitting the data in Fig. 2B with Eq. 1 (*n*=2). See also Fig. S2.
