# Supplementary material for: PTEN Hopping on the Cell Membrane Is Regulated via a Positively-Charged C2 Domain
Source: PLoS Comput Biol. 2014 Sep 11;10(9):e1003817. doi: 10.1371/journal.pcbi.1003817 (PMC4161299; doi:10.1371/journal.pcbi.1003817)
Supplement: Table S2 — Diffusion coefficients of wild-type PTEN and PTEN mutants in the two-component model. Parameters obtained from fitting the data in Fig. S3 with Eq. 2 (n = 2). The observable lowest D was ε 2/Δt, where ε is a standard deviation of measurement error calculated from the mean square displacement of the trajectories [20]. (DOCX) [file pcbi.1003817.s009.docx]

**Table S2**. Diffusion coefficients of wild-type PTEN and PTEN mutants in the two-component model.

| ***NAME*** | ***a*_1_** | ***a*_2_** | ***D*_1_ [μm^2^/s]** | ***D*_2_ [μm^2^/s]** |
| --- | --- | --- | --- | --- |
| PTEN | 0.924 | 0.034 | 0.026 | 0.423 |
| PTEN_1_ | 0.917 | 0.019 | 0.034 | 0.427 |
| PTEN_2_ | 0.943 | 0.016 | 0.045 | 0.518 |
| PTEN_3_ | 0.939 | 0.044 | 0.035 | 0.543 |
| PTEN_4_ | 0.900 | 0.074 | 0.020 | 0.593 |
| PTEN_5_ | 0.807 | 0.236 | 0.027 | 0.498 |
| PTEN_6_ | 0.839 | 0.178 | 0.021 | 0.508 |
| PTEN_7_ | 0.857 | 0.154 | 0.025 | 0.448 |
| PTEN_4_+Latrunculin A | 0.731 | 0.310 | 0.016 | 0.566 |

Parameters obtained from fitting the data in Fig. S3 with Eq. 2 (*n*=2). The observable lowest *D* was *ε*^2^/∆*t*, where *ε* is a standard deviation of measurement error calculated from the mean square displacement of the trajectories [20].
